# Culture-Independent and Culture-Dependent Characterization of the Black Soldier Fly Gut Microbiome Reveals a Large Proportion of Culturable Bacteria with Potential for Industrial Applications

**DOI:** 10.3390/microorganisms9081642

**Published:** 2021-07-31

**Authors:** Dorothee Tegtmeier, Sabine Hurka, Sanja Mihajlovic, Maren Bodenschatz, Stephanie Schlimbach, Andreas Vilcinskas

**Affiliations:** 1 Branch for Bioresources, Fraunhofer Institute for Molecular Biology and Applied Ecology (IME), 35392 Giessen, Germany; sanja.mihajlovic@ime.fraunhofer.de (S.M.); maren.bodenschatz@ime.fraunhofer.de (M.B.); stephanie.schlimbach@ime.fraunhofer.de (S.S.); 2 Institute for Insect Biotechnology, Justus Liebig University, 35392 Giessen, Germany; sabine.hurka@innere.med.uni-giessen.de

**Keywords:** black soldier fly, amplicon sequencing, 16S rRNA gene, culturable microbiome, genotyping, core microbiome, pathogen inhibition

## Abstract

Black soldier fly larvae (BSFL) are fast-growing, resilient insects that can break down a variety of organic substrates and convert them into valuable proteins and lipids for applications in the feed industry. Decomposition is mediated by an abundant and versatile gut microbiome, which has been studied for more than a decade. However, little is known about the phylogeny, properties and functions of bacterial isolates from the BSFL gut. We therefore characterized the BSFL gut microbiome in detail, evaluating bacterial diversity by culture-dependent methods and amplicon sequencing of the 16S rRNA gene. Redundant strains were identified by genomic fingerprinting and 105 non-redundant isolates were then tested for their ability to inhibit pathogens. We cultivated representatives of 26 genera, covering 47% of the families and 33% of the genera detected by amplicon sequencing. Among these isolates, we found several representatives of the most abundant genera: *Morganella*, *Enterococcus*, *Proteus* and *Providencia*. We also isolated diverse members of the less-abundant phylum *Actinobacteria*, and a novel genus of the order *Clostridiales*. We found that 15 of the isolates inhibited at least one of the tested pathogens, suggesting a role in helping to prevent colonization by pathogens in the gut. The resulting culture collection of unique BSFL gut bacteria provides a promising resource for multiple industrial applications.

## 1. Introduction

Black soldier fly (*Hermetia illucens*: Diptera, Stratiomyidae) larvae (BSFL) are useful for the bioconversion of organic materials into valuable compounds such as proteins, lipids and chitin, which can be used as food and feed additives and for the production of biopolymers for industrial and medical applications. BSFL protein can replace protein ingredients such as soy and fish meal in feed for several farmed and domestic animals. In 2017 the European Union approved BSFL for aquaculture feeding [1,2]. Conventionally, large amounts of fishmeal are used as feed for aquaculture, which causes several ecological problems such as marine overexploitation and entry of contaminants into the food chain. Therefore, alternative feed sources, such as BSFL, that are environmentally more friendly and sustainable, have come into research focus in the past years. The larvae are highly versatile because they can utilize diverse substrates, such as industrial side streams and waste products, with a remarkably efficient feed conversion ratio, making them one of the most economically important farmed insects [1,2,3]. Another advantage is their resilience against a variety of harmful substances, including aflatoxins [4], heavy metals [5], plant-derived natural insecticides [6] and pathogenic bacteria [7,8,9].

The degradation of diverse substrates by BSFL is mediated by an abundant gut microbiome, whose dynamic composition reflects the type of nutrients provided in the diet [6,10,11,12,13,14] and the developmental stage of the host [15,16]. Most studies of the BSFL gut microbiome involve next-generation sequencing, whereas cultivation-dependent studies that provide direct evidence of the properties and functions of gut bacterial isolates are still rare. Recent reports have addressed the BSFL microbiome as a source of microbes that can be added to the feed substrate to enhance larval growth performance (e.g., by producing fibrolytic enzymes) and the first such bacteria have been cultivated [17,18]. However, many gut microbes are difficult to cultivate because they require special nutrients, metabolic byproducts and other components, which they acquire as part of a complex metabolic network within the gut ecosystem [19,20,21]. In many insects e.g., termites, cockroaches and scarab beetle larvae, a large proportion of the gut microbiome is made up of obligate anaerobes that are adapted to the unique physiochemical conditions in the gut, such as a negative redox potential, extreme pH and high hydrogen partial pressure [22,23,24,25,26,27]. Therefore, most microbes found in the insect gut remain uncultivated and their potential industrial applications have yet to be realized.

The BSFL gut microbiome features core taxa from the families *Enterobacteriaceae*, *Enterococcaceae* and *Actinomycetaceae*, all of which are aerobes or facultative anaerobes. Furthermore, obligate anaerobic bacteria from the order *Clostridiales*, including the families *Lachnospiraceae*, *Ruminococcaceae* and *Clostridiaceae*, are also found in the BSFL gut microbiome, albeit mostly with low or variable relative abundances, depending on the diet [6,11,16]. The anaerobic bacterial community has been neglected so far and little is known about the culturability of the BSFL gut microbiome in general.

As well as breaking down organic material, the gut microbiome of many insects serves as a protective barrier to prevent colonization by pathogens and parasites [28,29,30,31,32,33,34]. BSFL can reduce the load of pathogens such as *Salmonella enterica* and *Escherichia coli* in feed substrates and even in manure [5,7,8,9,35]. The protection against pathogens is mediated by the production of antimicrobial peptides (AMPs) that are expressed by the BSFL in a diet-dependent manner [36]. Accordingly, BSFL extracts inhibit the bacterial pathogens *E. coli*, *Micrococcus luteus* and *Pseudomonas fluorescens* [36]. Beneficial microorganisms colonizing the gut and the feed substrate may provide colonization resistance and thus enhance the protection of BSFL against pathogens [6]. Such beneficial microbes can induce the production of AMPs by the host and enhance immunity or directly inhibit pathogens through the production of antibiotic metabolites [37,38]. However, the inhibition of pathogens by pure cultures of endogenous BSFL gut bacteria has not been investigated.

To gain more insight into the role of the BSFL microbiome, we evaluated its bacterial diversity and its culturability using a broad sampling approach over several rearing cycles. We combined 16S rRNA amplicon sequencing with a cultivation-dependent approach using a variety of selective media as well as aerobic and anaerobic cultivation techniques and evaluated the redundancy of the bacterial gut isolates by genomic fingerprinting. Finally, selected strains were tested for their ability to inhibit pathogens.

## 2. Materials and Methods

### 2.1. Black Soldier Fly Breeding

Black soldier flies were obtained from Bio.S Biogas (Grimma, Germany) and reared as previously described [6]. Briefly, eggs were harvested using plastic spatulas, and 200 mg (approximately 9000 eggs) were placed in a plastic box (19.5 cm × 16.5 cm × 9.5 cm) and sprayed with water before closing the lid. Larvae were reared in a climate room at 27 ± 1 °C and 65 ± 5% relative humidity in the dark. When 50% of the larvae had hatched, the lid was replaced with a fine mesh and Golddott Eierglück ground chicken feed (Raiffeisen, Münster, Germany) was provided for nutrition. Additional water and feed were provided *ad libitum* until larvae reached the prepupal stage. The humidity of the substrate was adjusted to 70%. Disposable nitrile gloves were worn throughout. BSFL were reared continuously under the same conditions.

### 2.2. Analysis of the BSFL Microbial Community by Amplicon Sequencing

BSFL were collected from three different rearing cycles and frozen at −20 °C. The larval instar was determined according to Barros et al. [39]. Prior to dissection, 5th instar larvae (L5) were separated into two groups: G1 (200–250 mg per larva) and G2 (300–350 mg per larva). The larvae were washed with 70% ethanol and rinsed with sterile water. Whole guts were dissected with sterile forceps under a stereomicroscope and were frozen at –20 °C ready for DNA extraction. Forceps were washed with sterile water and ethanol after each dissection.

Pools of three guts were disrupted by bead beating in a FastPrep-24 (MP Biomedicals, Solon, OH, USA) for 2 × 45 s at 6.5 m·s^−1^. DNA was extracted with the NucleoSpin Soil kit (Macherey-Nagel, Düren, Germany) according to the manufacturer’s instructions and checked for quantity and purity by spectrophotometry on a Take 3 plate reader (BioTek Instruments, Winooski, VT, USA). Two high-quality DNA extracts were pooled in equimolar amounts for each sample. For amplicon sequencing, we generated three samples of G1 and three samples of G2.

Libraries were prepared and sequenced by LGC Genomics (Berlin, Germany) using primers U341F (5′-CCT AYG GGR BGC ASC AG-3′) and U806R (5′-GGA CTA CNN GGG TAT CTA AT-3′) [40] to amplify variable region V3–V4 of the 16S rRNA gene of bacteria and archaea. The libraries were sequenced on an Illumina (San Diego, CA, USA) MiSeq V3 to generate approximately 100,000 paired-end reads per sample, with a read length of 300 bp. Samples were multiplexed and pooled for sequencing.

Sample demultiplexing and the clipping of adapters and primers were carried out by LGC Genomics using bcl2fastq 2.17.1.14 (Illumina). Reads were analyzed using QIIME 2020.6 [41]. We used the DADA2 plugin [42] for error correction, quality control, filtering of chimeric sequences and the creation of an amplicon sequence variant (ASV) table listing the number of sequences for each observed ASV per sample.

Taxonomic classification was carried out using a self-trained naïve Bayes classifier based on SILVA SSU Ref NR 99 138.1 [43]. We used RESCRIPt 2020.11.1.dev0 for building [44], including trimming the reference sequences to the specific region targeted by the amplicon primers [45]. Confidence for classification was set to 0.7 as recommended [46]. *Flavobacterium cloacae* was manually renamed to *Myroides cloacae* [47,48]. Furthermore, each ASV was classified by running a BLAST search against the NCBI nucleotide database (https://www.ncbi.nlm.nih.gov/ (accessed on 22 April 2021)) in Geneious 10.2.6 (https://www.geneious.com). The following thresholds were used for taxon assignment: species 98.7%, genus 96.4%, family 90.1%, order 81.0% and class 70.0% [49,50,51]. Alpha diversity was based on Faith’s phylogenetic diversity [52] and the observed ASVs. For statistics and graphics, we used Microsoft Excel and R 4.1.0 (https://www.r-project.org) with packages qiime2R, tidyverse, plyr, VennDiagram, ggrepel, phylotools and ggpubr.

### 2.3. Cultivation and Isolation of Gut Bacteria

BSFL in the weight range of 200–350 mg (L5), collected from four different rearing cycles, were washed with 70% ethanol and sterile water, and the guts were dissected with sterile forceps under a stereomicroscope. Five guts per rearing cycle were pooled and transferred to a sterile 16 mL glass tube containing 2 mm glass beads and 5 mL basal medium, and were homogenized by vortexing for 10 min. The isolation strategy described below was developed to target endogenous BSFL gut bacteria, particularly those representing the core microbiome [6,12,15,16].

Serial dilution series of the gut homogenates were prepared in 16 mL glass tubes containing phosphate-buffered saline (PBS), anaerobe basal broth (Oxoid, Wesel, Germany), carboxymethyl cellulose (CMC) medium (modified ATCC medium 2720) or *Azotobacter* medium (DSMZ medium 3). We plated 200 µL of each dilution onto solid media. The dilution series in PBS was plated onto cellulose agar (ATCC medium 907), 100% casein soy peptone (CASO) agar, 10% CASO agar and lysogeny broth (LB) agar, targeting *Enterobacteriaceae* [53] and actinomycetes [54]. The dilution series in *Azotobacter* medium was plated onto *Azotobacter* agar, which targets nitrogen-fixing bacteria. The dilution series in CMC medium (in glass tubes gassed with helium) was plated onto CMC agar, which targets obligate anaerobic *Clostridiaceae*, *Ruminococcaceae* and facultative anaerobic *Enterobacteriaceae* [55]. CMC plates were incubated under air or under anoxic conditions in an anoxic jar with AnaeroGen gas packs (Oxoid). The dilution series in anaerobe basal broth was plated onto anaerobe basal agar, which targets a variety of obligate and facultative anaerobic gut bacteria. Plates were incubated under anoxic conditions (as described above). Dilutions at 1:10^−4^–10^−8^ were plated onto rich media (100% CASO, LB and anaerobe basal agar) and dilutions at 1:10^−2^–10^−6^ were plated onto selective media (10% CASO, CMC, cellulose and *Azotobacter* agar).

For the isolation of spore-forming *Bacillus* and *Clostridia* species, 2-week-old cultures, grown in CMC medium, were pasteurized by incubation at 90 °C for 10 min before streaking on CMC agar, CASO agar (100%) and anaerobe basal agar, followed by aerobic and anaerobic incubation. All cultures were incubated at 27 °C (the same temperature used for BSFL breeding).

Single colonies were picked with a sterile inoculation loop and separated (at least three transfers) until the cultures were pure. Culture purity was confirmed by viewing under an S9i stereomicroscope (Leica Microsystems, Wetzlar, Germany) and, when necessary, a DM 2500 LED phase contrast microscope (Leica Microsystems). Cultures were preserved by preparing duplicate stocks in cryo-vials containing a rich growth medium with anti-freeze additive (Roti-Store, Carl Roth), followed by storage at −80 °C.

### 2.4. Phylogenetic Classification of Isolates and Comparison with Amplicon Sequences

DNA was routinely extracted from pure cultures by incubating cell material in 0.2% sodium dodecylsulfate (SDS) at 95 °C for 10 min and diluting 1:10 in nuclease-free water. For Gram-positive species, cells were disrupted by bead beating in a FastPrep-24, followed by extraction with the NucleoSpin Soil kit, as described above. The 16S rRNA genes were amplified with *Bacteria*-specific primers as previously described [56] and sequenced using the Sanger method by Eurofins Genomics (Ebersberg, Germany) or Microsynth Seqlab (Göttingen, Germany). 

Sequences were evaluated, trimmed and assembled using Geneious 10.2.6. Taxonomic classification of each sequence was applied using a naïve Bayes classifier based on SILVA 138.1 and by running a BLAST search against the NCBI nucleotide database (accessed on 03 May 2021) as described above. 

Comparing the SILVA and NCBI classification allowed us to assign the genus of strain 01-109 as *Bordetella* in our SILVA data, with online support from SINA 1.2.11 (https://www.arb-silva.de/aligner/). All sequences were aligned in Geneious 10.2.6 to sequences of the closest related type strain using ClustalW (with default parameters). The alignment was checked manually and corrected if necessary. Phylogenetic trees and distance matrices were calculated using MEGA 7.0.268 (https://www.megasoftware.net) with the maximum-likelihood method in the Tamura-Nei model [57] with 1000 bootstrap replications and the same thresholds for novel taxa as described above [49,50,51].

VSEARCH 2.17.0 was used for global pairwise alignment to compare all 208 ASVs to our Sanger sequences. All matches with an identity of 100% were retained. For further analysis, ASVs were also designated as matched or unmatched.

### 2.5. Genomic Fingerprinting

Fingerprinting was achieved by BOX-PCR and GTG_5_-PCR. The conditions for BOX PCR are described elsewhere [58]. GTG_5_-PCR was carried out in a total volume of 25 µL including 1 µL of DNA template, 0.625 U of Dream Taq Polymerase, 1 × Dream Taq buffer, 200 µM of each dNTP and 1 µM GTG_5_ primer (5′-GTG GTG GTG GTG GTG-3′). The cycling conditions began with denaturation at 95 °C for 3 min followed by 30 cycles of 95 °C for 30 s, 40 °C for 1 min and 65 °C for 8 min and a final extension step at 65 °C for 16 min. Genomic fingerprinting patterns were analyzed using Lab Chip GX Touch HT microfluidics technology with the 5K DNA Assay (cat. no. CLS760675; PerkinElmer, Waltham, MA, USA) and GelCompare II 6.5 (Applied Maths, Sint-Martens-Latem, Belgium) for data interpretation. A dendrogram was constructed based on Dice similarity matrix data by applying the unweighted pair group method with arithmetic average (UPGMA) cluster analysis.

### 2.6. Screening for Antimicrobial Activity

Non-redundant bacterial isolates were selected based on the fingerprinting results. Selected isolates were tested in duplicate for their activity against the common pathogens *E. coli* K12, *Staphylococcus aureus* DSM 799 and *Pseudomonas aeruginosa* DSM1117 in an inhibition zone assay on LB agar plates containing each pathogen. Overnight cultures were prepared by inoculating 100 mL LB medium (in Erlenmeyer flasks) with 100 µL of a 24 h-old pre-culture of each pathogen, followed by incubation at 37 °C and shaking at 200 rpm for 24 h. Hand-hot LB agar was inoculated and mixed with 0.1% of the overnight culture and poured into square Petri dishes (120 × 120 mm). Once the agar had set, the BSFL gut isolates were applied to the agar with an inoculation loop (up to 48 isolates per plate) and incubated for 48 h at 27 °C under aerobic conditions. Obligate anaerobic strains were not included in the test. After incubation, activity against the pathogens was evaluated by measuring zones of inhibition, indicated by the full or partial clearance of agar turbidity.

## 3. Results

### 3.1. Analysis of the BSF Microbial Community by Amplicon Sequencing

**Figure 1:**
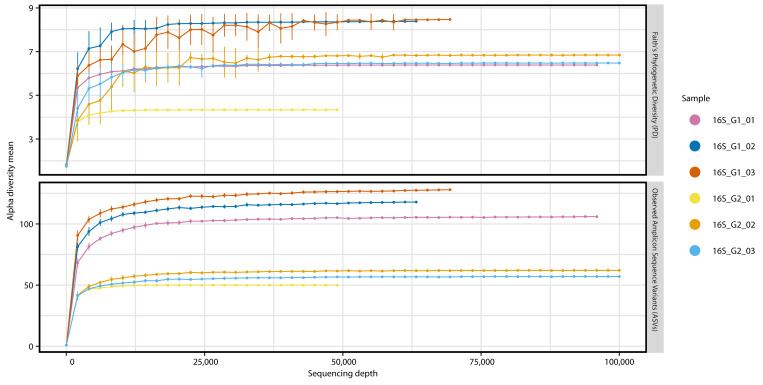
Rarefaction curves of 16S rRNA gene sequencing for all samples. All curves reached a plateau, showing that greater sequencing depth would not influence the microbial diversity found in the sequencing data (Faith’s phylogenetic diversity and observed amplicon sequence variants). Standard deviations are shown as bars.

We generated 695,023 paired-end reads from six samples (containing DNA from 36 BSFL guts). Quality control, removal of chimeric sequences and merging of paired reads delivered 523,594 final reads with an average length of 422 bp. The number of reads per sample ranged from 49,479 to 140,464. Rarefaction curves showed that the number of ASVs reached a plateau for all samples, confirming the sequencing depth was sufficient (Figure 1).

We identified 213 unique ASVs across all six samples using DADA2 and our naïve Bayes classifier based on SILVA. Five ASVs were only specified as domain Bacteria or unassigned. BLAST queries against the NCBI nucleotide database identified these ASVs as eukaryotic sequences assigned to *Hermetia illucens*, which led us to remove them from the dataset. The remaining 208 ASVs were assigned to the domain Bacteria. No ASVs were assigned to domain Archaea. Taxonomic labels to the genus level could be assigned for 179 ASVs, and to the species level for 46 ASVs. Therefore, we focused on the genus and family levels for further analysis.

In general, classifications of the ASVs using our SILVA-based naïve Bayes classifier were similar to the BLAST results against the NCBI nucleotide database. Classifications of taxonomic order, family and genus were similar in both databases for 183, 190 and 158 ASVs, respectively (taxonomic reassignments not taken into account). Detailed classifications from phylum to genus level and the corresponding ASV counts are shown in Supplementary Table S1.

For subsequent analysis, we used the classifications based on SILVA. The ASVs from all six samples were assigned to the phyla *Proteobacteria* (56.7%), *Firmicutes* (33.5%), *Actinobacteria* (6.2%) and *Bacteroidetes* (3.6%) as shown in Supplementary Table S1.

**Figure 2:**
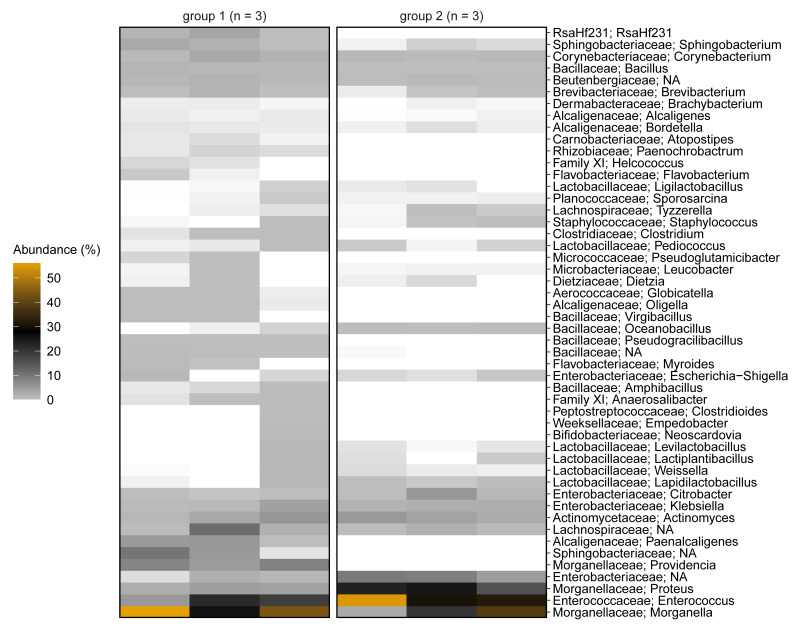
Heat map showing the bacterial communities in the BSFL guts representing group 1 (G1) and group 2 (G2) larvae, based on 16S rRNA gene amplicon sequencing. The relative abundances of ASV counts are collapsed to the genus level for all replicates. Family assignments are prefixed. Percentage relative abundance is color-coded from light gray, through black, to orange, the last representing the highest abundance. Families depicted in white were not detected in the sample. Only the top 50 genera are shown, measured by the mean counts per genus level over all samples. Genera Clostridium sensu stricto 1, 15 and 18 are collapsed to the genus *Clostridium*. Genera with undefined family and genus are not shown.

Both larval groups (G1 and G2) showed a very high abundance of *Morganellaceae* (52.5% and 41.9%, respectively), which were assigned to the genera *Morganella*, *Proteus* and *Providencia*. Furthermore, *Enterococcaceae* (all assigned to the genus *Enterococcus*; 15.1% in G1 and 39.1% in G2), *Actinomycetaceae* (all assigned to the genus *Actinomyces*; 3.2% in G1 and 3.8% in G2), *Lachnospiraceae* (4.7% in G1 and 1.1% in G2) and *Enterobacteriaceae* (3.4% in G1 and 11.7% in G2; assigned to the genera *Citrobacter*, *Klebsiella* and *Escherichia*-*Shigella*), were found in both groups (Figure 2).

We also found differences between the two larval groups, including the presence of *Clostridiaceae*, *Peptostreptococcales*-*Tissierellales* (Family XI), *Candidatus* RsaHF231 and genus *Paenochrobactrum* in all replicates of G1 and their complete absence in all replicates of G2 (Figure 2 and Supplementary Table S1). Alpha diversity was generally higher in G1 when compared to G2: The count of observed ASVs were higher in all replicates of G1 and Faith’s phylogenetic diversity was higher in two replicates of G1 (Figure 1).

### 3.2. Isolation, Identification and Phylogenetic Analysis of Bacterial Isolates from BSFL Guts

**Figure 3:**
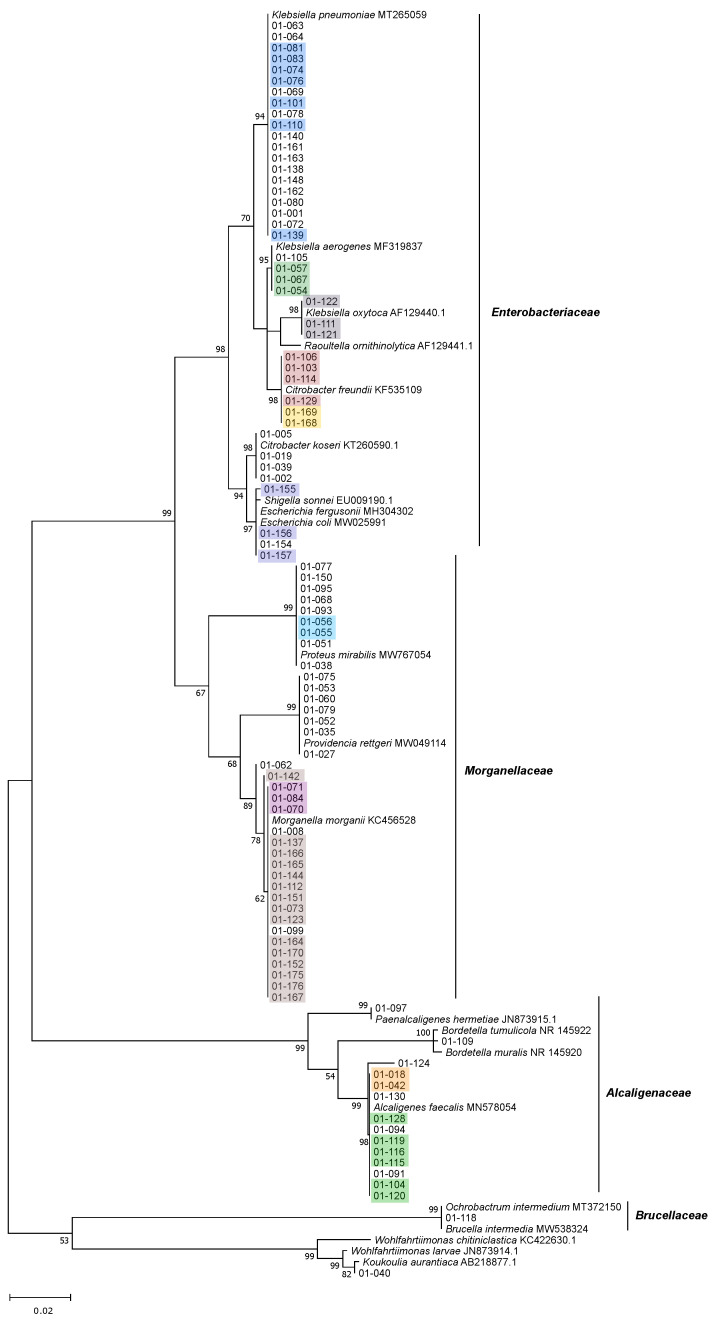
Unrooted maximum-likelihood tree of all BSFL gut isolates belonging to the phylum *Proteobacteria* represented, along with the closest related type strains and other relevant strains. Phylogenetic analysis was based on the unambiguous alignment (1306 sequence positions) of 16S rRNA gene sequences. GenBank accession numbers are included. Numbers at nodes indicate bootstrap values, based on 1000 replications. Identical isolates based on fingerprinting results (see Supplementary Figure S1) are marked with the same color.

**Figure 4:**
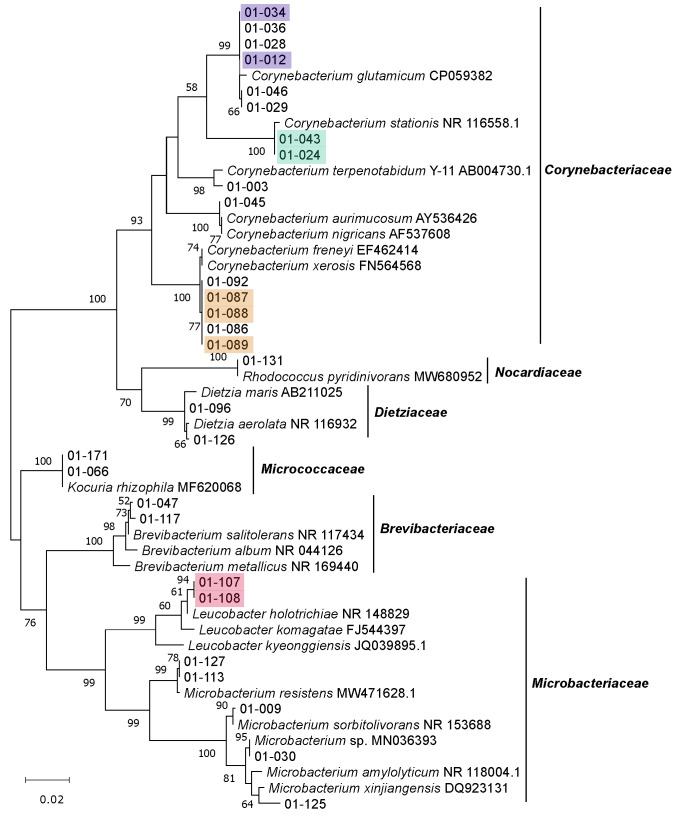
Unrooted maximum-likelihood tree of isolates belonging to the phylum *Actinobacteria*, shown, along with the closest related type strains and other relevant strains. Phylogenetic analysis was based on the unambiguous alignment (1274 sequence positions) of 16S rRNA gene sequences. GenBank accession numbers are included. Numbers at nodes indicate bootstrap values, based on 1000 replications. Identical isolates based on fingerprinting results (see Supplementary Figure S1) are marked with the same color.

**Figure 5:**
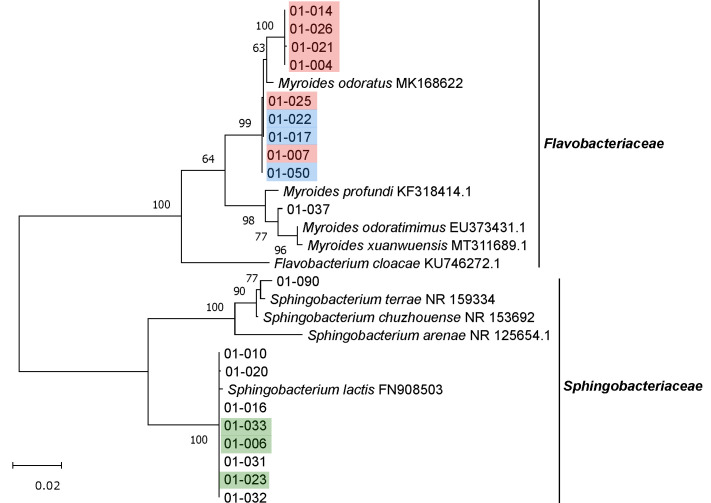
Unrooted maximum-likelihood tree of isolates belonging to the phylum *Bacteroidetes*, shown along with the closest related type strains and other relevant strains. Phylogenetic analysis was based on the unambiguous alignment (1171 sequence positions) of 16S rRNA gene sequences. GenBank accession numbers are included. Numbers at nodes indicate bootstrap values, based on 1000 replications. Identical isolates based on fingerprinting results (see Supplementary Figure S1) are marked with the same color.

**Figure 6:**
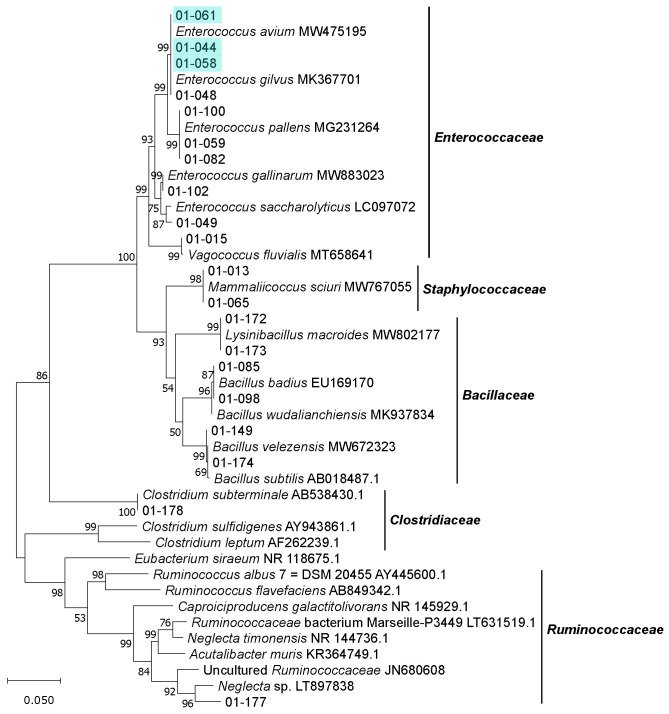
Unrooted maximum-likelihood tree of isolates belonging to the phylum *Firmicutes*, shown along with the closest related type strains and other relevant cultured and uncultured bacteria. Phylogenetic analysis is based on the unambiguous alignment (1306 sequence positions) of 16S rRNA gene sequences. GenBank accession numbers are included. Numbers at nodes indicate bootstrap values, based on 1000 replications. Identical isolates based on fingerprinting results (see Supplementary Figure S1) are marked with the same color.

We isolated 162 bacterial strains from the BSFL gut samples, 160 of which yielded full-length 16S rRNA sequences, following the assembly of the forward and reverse reads, to a total read length of 1300 bp. For two isolates, 01-047 and 01-155, we did not obtain the reverse read, even after several sequencing attempts, so we used the forward read alone (1108 and 955 bp, respectively) for further analysis. The detailed results of the classifications based on SILVA and BLAST queries against the NCBI nucleotide database are shown in Supplementary Table S2. Based on the pairwise comparison of 16S rRNA sequences similarities, we identified 62 isolates with unique sequences, indicating that the sequences of 100 isolates (61.7%) are redundant (Figure 3, Figure 4, Figure 5 and Figure 6 and Supplementary Table S3).

The isolates belonged to the phyla *Proteobacteria* (94 isolates), *Actinobacteria* (29 isolates), *Firmicutes* (20 isolates) and *Bacteroidetes* (19 isolates), which were also the four major phyla detected by amplicon sequencing. We found no representatives of any other phyla (Figure 3, Figure 4, Figure 5 and Figure 6).

Within the most prominent phylum (*Proteobacteria)*, 41 isolates belonged to the family *Enterobacteriaceae* and 37 to the family *Morganellaceae* (formerly *Enterobacteriaceae*) and were assigned to the genera *Klebsiella* (27 isolates)*, Morganella* (21 isolates)*, Citrobacter* (10 isolates), *Proteus* (nine isolates), *Providencia* (seven isolates) and *Escherichia*-*Shigella* (four isolates). All *Enterobacteriaceae* and *Morganellaceae* were isolated from a variety of different media, including 100% CASO agar, LB agar, anaerobe basal agar and CMC agar. Four *Klebsiella* strains were isolated from *Azotobacter* agar.

Fourteen isolates belonged to the family *Alcaligenaceae* (assigned to the genera *Alcaligenes*, *Paenalcaligenes* and *Bordetella*) and 12 of them were isolated from CMC agar. One isolate was assigned to the family *Brucellaceae* (and to the genus *Brucella*, formerly *Ochrobactrum*). Furthermore, one isolate was assigned to the genus *Wohlfahrtiimonas* (Gammaproteobacteria incertae sedis, not assigned to a family) showing a close relationship to *Wohlfahrtiimonas larvae* (99.2% sequence identity), a strain previously isolated form the BSFL gut [59]. Its closest relative is a strain named *Koukoulia aurantiaca*, which has not yet been described (Figure 3).

Within the phylum *Actinobacteria*, 15 isolates were assigned to the family *Corynebacteriaceae* (all representing the genus *Corynebacterium*), seven isolates to the family *Microbacteriaceae* (genus *Microbacterium* and *Leucobacter*), two to the family *Dietziaceae* (both genus *Dietzia*), two to the family *Micrococcaceae* (both genus *Kocuria*), two to the family *Brevibacteriaceae* (both genus *Brevibacterium*) and one to the family *Nocardiaceae* (genus *Rhodococcus*) (Figure 4). All *Actinobacteria* isolates (except the two strains assigned to the genus *Kocuria*) were obtained from either CMC agar or 10% CASO agar plates.

Within the phylum *Bacteroidetes*, 10 isolates belonged to the family *Flavobacteriaceae* (all genus *Myroides*) and nine to the family of *Sphingobacteriaceae* (all *Sphingobacterium lactis*) (Figure 5). Eighteen of the isolates were obtained from cultures grown on 10% CASO agar. One strain (01-090) was isolated on cellulose agar and was assigned to *Sphingobacterium terrae* or *Sphingobacterium chuzhouense* with 99.1% sequence identity (Supplementary Table S3).

Within the phylum *Firmicutes*, 10 isolates belonged to the family *Enterococcaceae* (genus *Enterococcus* and *Vagococcus*). A further six isolates were assigned to the family *Bacillaceae* (genus *Bacillus* and *Lysinibacillus*) and were all cultivated on cellulose or CMC media. Three of the isolated *Bacillaceae* strains were obtained from pasteurized cultures. One isolate belonged to the family *Staphylococcaceae* (genus *Mammaliicoccus,* formerly *Staphylococcus*).

Furthermore, two strains assigned to the order *Clostridiales* were isolated from pasteurized cultures (CMC medium). Strain 01-178 was assigned to the genus *Clostridium*, whereas strain 01-177 could not be assigned to any genus and its family status was also not fully elucidated. Its closest relative is *Neglecta* sp., a strain that has yet to be described. The genus *Neglecta* is represented by *Neglecta timonensis*, which has been assigned to the order *Clostridiales* [60] but not yet to any family. The closest validly described relative of strain 01-177 is *Acutalibacter muris* with 90.4% sequence identity (Figure 6 and Supplementary Table S3). A. *muris* was assigned to the family *Ruminococcaceae* [61]. The affiliation of strain 01-177 to the family *Ruminococcaceae* is also supported by its close relationship to other (thus far uncultured) members of the same family and its high bootstrap values.

Most of the isolates were obtained from cultures grown under atmospheric oxygen and are therefore aerobes or facultative anaerobes. Nineteen isolates were obtained from cultures grown under anoxic conditions, but 17 of them also grew under atmospheric oxygen and are considered to be facultative anaerobes or at least aerotolerant anaerobes. Most of the facultative anaerobe isolates belonged to the phylum *Proteobacteria* and were assigned to the genera *Proteus*, *Providencia*, *Klebsiella*, *Morganella* and *Escherichia-Shigella*. Three facultative or aerotolerant anaerobic isolates belonged to the phylum *Firmicutes* and were assigned to the genera *Enterococcus* and *Staphylococcus*. The two strains of *Clostridiales* grew only under anoxic conditions and were considered to be obligate anaerobes.

### 3.3. The Culturable BSFL Gut Microbial Community

**Figure 7:**
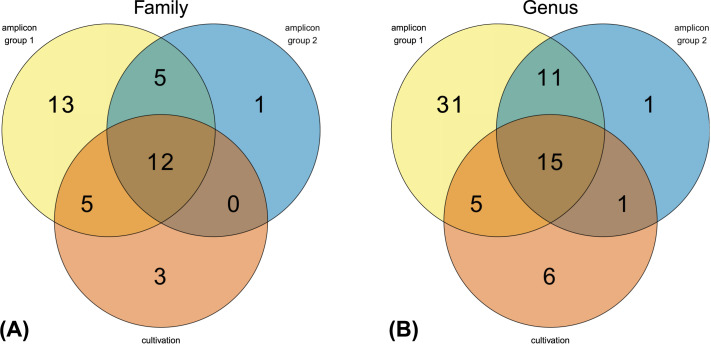
Venn diagram showing shared (**A**) families and (**B**) genera identified in the amplicon data (group 1, group 2) and cultivation-dependent approach (Sanger sequencing). Only one representative per group and (**A**) family or (**B**) genus was counted. Undefined taxa were excluded.

Amplicon sequencing revealed 80 different genera, 64 of which were assigned to validly described genera and the remaining 16 to genera with no cultured representatives (NA). We detected 62 different genera in larval group 1 (G1) and 28 different genera in larval group 2 (G2) with 26 genera in common, indicating that all but two genera detected in G2 were also present in G1 (Figure 7).

The 161 isolates obtained from BSFL guts from the cultivation approach could be assigned to 20 different families and 27 different genera. One isolate (01-177) was found to represent a novel genus of *Clostridiales*. 48.6% of the families present in G1 and 66.7% of the families present in G2 were covered by corresponding representatives obtained by the cultivation approach. On genus level 32.3% of G1 and 57.1% of G2 were covered by cultivated representatives (Figure 7).

Overall, 17 of the isolated families and 21 of the isolated genera were represented in the amplicon data. Therefore, representatives of 47.2% of all families and 32.8% of all genera found in the amplicon data were isolated with our cultivation approach. Three isolates were not found by amplicon sequencing on genus level but belong to families, for which we found at least one representative in the amplicon data. Vice versa 85.0% of the families and 77.8% of the genera for which we isolated representatives are contained in the amplicon data. 

Most of the amplicon sequences (54.8%) and the Sanger sequences of the isolates (48.1%) were assigned to the families *Morganellaceae* and *Enterobacteriaceae.* Several members of other abundant families found by amplicon sequencing (*Enterococcaceae*, *Sphingobacteriaceae* and *Alcaligenaceae*) were also isolated from the gut. We isolated representatives of nine of the 10 most abundant genera (≥1% relative abundance found in the amplicon data), including the genera *Morganella*, *Enterococcus*, *Proteus*, *Providencia*, *Sphingobacterium*, *Klebsiella*, *Paenalcaligenes*, *Corynebacterium* and *Citrobacter*. Seven of these most abundant genera were detected in all replicates of G1 and G2. Only *Providencia* and *Paenalcaligenes* were absent from G2 but present in all replicates of G1. Furthermore, several representatives of genera with a low relative abundance in the amplicon data (0.001–0.7%), including *Bacillus*, *Brevibacterium*, *Escherichia*-*Shigella*, *Myroides*, *Staphylococcus*, *Clostridium*, *Leucobacter*, *Dietzia*, *Bordetella*, *Alcaligenes*, *Vagococcus* and *Lysinibacillus*, were successfully isolated using our approach. With the exception of *Lysinibacillus*, *Vagococcus* and *Myroides*, these genera were represented in at least three of the six BSFL gut samples. 

The only abundant taxa (≥1% relative abundance in the amplicon data) that escaped our isolation strategy were members of the family *Lachnospiraceae* (mostly uncultured representatives) and the genus *Actinomyces*. Furthermore, representatives of *Candidatus* RsaHf231, which was only detected in G1, were not targeted by our isolation approach. The remaining 43 genera that we did not isolate were present with a very low relative abundance in the amplicon data (0.0002–0.3%). The 13 least abundant taxa were only present in one of the six BSFL gut samples. However, members of families that were not detected by amplicon sequencing (including family *Nocardiaceae* and unclassified *Gammaproteobacteria* from genus *Wohlfahrtiimonas*) were obtained by the cultivation approach, but each family was only represented by a single isolate (Supplementary Tables S1 and S2).

**Figure 8:**
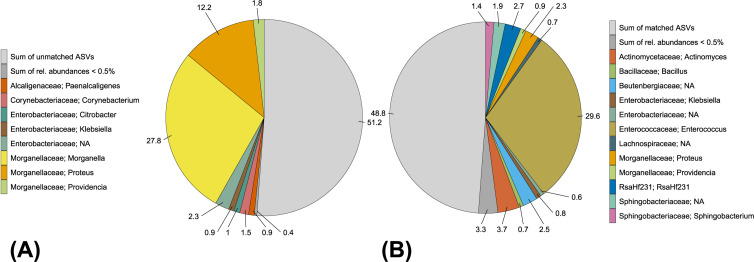
Bacterial composition at the genus level in the amplicon data depending on ASVs matching or not matching Sanger sequences with 100% identity. Numbers indicate percent relative abundance. Genera with <0.5% relative abundance are consolidated. (**A**) Distribution of 23 ASVs aligned to 92 Sanger sequences (matched). (**B**) Distribution of 185 ASVs not aligned to our Sanger sequences (unmatched).

Aligning all 208 ASVs against the 162 Sanger sequences using VSEARCH identified 23 ASVs that matched 92 Sanger sequences with 100% identity. These 23 ASVs covered 48.8% of the relative abundance in the amplicon data (Figure 8).

### 3.4. Genomic Fingerprinting of Bacterial Isolates from the Cultivation Approach

We were able to fingerprint 157 of the 162 isolates by BOX-PCR and a further three by GTG_5_-PCR (01-048, 01-049 and 01-098), leaving only two strains without a genomic fingerprint (01-009 and 01-065). We identified 55 redundant strains (34.4% overall redundancy) based on the fingerprint pattern, leaving 105 putatively unique strains. The detailed fingerprinting results showing genetic relatedness of the isolates based on BOX and GTG5-PCR banding patterns are shown in Supplementary Figures S1 and S2, respectively. Most redundancy was detected in the phyla *Bacteroidetes* (47.4%) and *Proteobacteria* (39.4%). Lower redundancy was found in the phyla *Actinobacteria* (17.2%) and *Firmicutes* (10.0%). Many strains that were potentially identical based on pairwise comparisons of 16S rRNA sequences were found to be non-redundant based on fingerprinting data (Figure 3, Figure 4, Figure 5 and Figure 6 and Supplementary Figure S1).

### 3.5. Screening for Antimicrobial Activity among the Isolates from the Cultivation Approach

We found that 15 of the 105 isolates approved by fingerprinting showed activity against at least one of the tested pathogens listed in Table 1. Four isolates showed activity against *S. aureus*, five against *E. coli* and 11 against *P. aeruginosa*. Strain 01-149 (assigned to *Bacillus velezensis*) inhibited all three afore mentioned pathogens. Activity against *E. coli* was further observed for strains 01-018 and 01-124 (assigned to *Alcaligenes faecalis*) as well as for strains 01-054 and 01-105 (assigned to *Klebsiella aerogenes*). Activity against *S. aureus* was further observed for strains 01-018 and 01-115 (assigned to *Alcaligenes faecalis*) and 01-040 (assigned to *Wohlfahrtiimonas larvae*). Finally, activity against *P. aeruginosa* was observed for strain 01-008 (assigned to *Morganella morganii*) and strains 01-040, 01-124 and 01-149. Strains 01-027, 01-035, 01-052, 01-053, 01-060 and 01-079 (all assigned to *Providencia rettgeri*) and strain 01-003 (assigned to *Corynebacterium terpenotabidum*) achieved partial clearance and are considered weakly active against *P. aeruginosa* (Table 1).

## 4. Discussion

### 4.1. Amplicon Sequencing Versus Cultivation-Dependent Microbiome Analysis

Approximately 99% of bacterial and archaeal species on earth have yet to be cultured [62] or might not be culturable at all, and are thus described as “microbial dark matter” [63]. Accordingly, culture-dependent methods often produce biased results that depend on the media and cultivation techniques. Although, next-generation sequencing methods can also be afflicted by a certain bias (e.g., by DNA isolation process, the choice of 16S rRNA region and PCR efficiency), they often provide a more realistic view of microbial communities. Furthermore, amplicon sequencing is less time-consuming than cultivation using a variety of selective and non-selective media. It also allows multiple samples to be analyzed in parallel, which is not possible when using the cultivation approach described herein. We therefore used amplicon sequencing to analyze six samples of pooled guts from two different larval weight groups (G1 and G2). Although, we found many similarities in community composition between the two groups, we also found that certain taxa present in all replicates of G1 were completely missing in G2. The resulting lower alpha diversity in G2 indicates that the microbial community can vary depending on larval weight or sampling day. Such variances were also found by Klammsteiner et al. [12]. The overall community composition in both G1 and G2 showed many similarities to that of BSFL reared on chicken feed in previous studies e.g., the presence of *Enterobacteriaceae*, *Morganellaceae*, *Actinomycetaceae* and *Enterococcaceae* [6,12,16], corroborating their designation as core-taxa.

However, by using short-read next-generation sequencing techniques, many species remain unidentified when it comes to lower taxonomic ranks and accordingly we found that only 46 of 208 ASVs could be assigned at the species level. Therefore, culture-dependent methods are an important amendment for characterizing the insect gut microbiome because this allows the sequencing of the full 16S rRNA gene, genomic fingerprinting and whole-genome sequencing of the isolates, facilitating accurate classification to the species level as well as phylogenetic analysis. Furthermore, industrial applications of gut bacteria rely on their culturability. Physiological and genomic properties of the isolates can be studied in detail and might reveal undiscovered possibilities.

Culturing the insect gut microbiome is often challenging because many insects harbor specialized microbes that co-evolved with their host [27,64] and are fully adapted to physiochemical conditions in the gut, including its negative redox potential. In contrast, the BSFL gut microbiome is versatile, with a flexible core community that offers a promising source for isolating diverse microbes [14,65]. Most of the taxa found by amplicon sequencing were facultative anaerobes, allowing faster growth and a less fastidious cultivation approach compared to obligate anaerobes, which is also an advantage for industrial applications.

We were able to cultivate representatives of at least 47% of the families and 33% of the genera detected by amplicon sequencing. The most frequently isolated genera (*Klebsiella*, *Morganella*, *Providencia* and *Escherichia*) were also isolated in other recent studies of BSFL [17,18]. *Morganella* and *Providencia* were also found by amplicon sequencing in several studies using a variety of diets [6,13,15,66] indicating they belong to the BSFL core microbiome. Furthermore, the genera *Alcaligenes*, *Enterococcus* and *Bacillus* isolated from BSFL in earlier work [17] were also isolated in our study and identified by amplicon sequencing. Two of our isolates (*Paenalcaligenes hermetiae* and *Wohlfahrtiimonas larvae*) were previously isolated from BSFL guts [59,67], although the latter was not detected among our amplicon sequences.

Notably, we isolated representatives of nine of the 10 most abundant genera found by amplicon sequencing and most of the taxa that escaped our cultivation approach were those with a low relative abundance. One potential reason for the lower diversity covered by the cultivation approach is the smaller sample size compared to the amplicon sequencing approach. Furthermore, a cultivation approach might not cover such a depth as provided by amplicon sequencing. Next-generation sequencing allows the detection of a single bacterium, which, possibly, cannot be isolated from the bulk of gut bacteria. Therefore, we expected to isolate primarily members of the core microbiome (autochthonous bacteria) and to overlook some infrequently occurring bacteria. The 13 least abundant taxa were only present in one of six BSFL gut samples, indicating they are allochthonous bacteria (possibly transient) and not common members of the BSFL gut microbiome, thus reducing the chance of isolation. However, we were also unable to isolate some frequently occurring representatives of the family *Lachnospiraceae*, the genus *Actinomyces* (detected in all replicates of G1 and G2) and *Candidatus* RsaHf231 (detected in all replicates of G1). The family *Lachnospiraceae* and several members of the genus *Actinomyces* are fastidious anaerobic bacteria, which often require high levels of carbon dioxide and various supplements possibly not included in our media [68,69,70]. No members of *Candidatus* RsaHf231 have been cultivated thus far, so it would be challenging to select appropriate conditions in the absence of information about their physiology and growth requirements. This lineage was first detected in termite guts [71] and was recently also found in BSFL guts [5]. A possibility that would grant first insights into the metabolism and possible requirements of this group is the generation of metagenomic data from BSFL guts of G1 and the assemblage of genomes of this lineage.

Many of the taxa we isolated, including several species of *Corynebacterium*, *Sphingobacterium*, *Brevibacterium*, *Dietzia*, *Microbacterium* and species belonging to the genera *Kocuria* and *Rhodococcus*, have not been isolated from BSFL before. We also obtained two obligate anaerobic strains belonging to the order *Clostridiales*, for which representatives were isolated for the first time from BSFL guts. One of these isolates (strain 01-177) represents a novel genus, indicated by the 16S rRNA sequence identity of only 90.4% compared to its closest relative (*Acutalibacter muris*) and the phylogenetic distance to other species in the family *Ruminococcaceae*. The low sequence identity is close to the threshold to infer a novel family [49,50], so strain 01-177 is an interesting candidate for detailed physiological and genomic characterization to fully elucidate its taxonomic affiliation and to provide novel insights into the family *Ruminococcaceae* or the order *Clostridiales*.

The use of various selective media also provided the first impressions of the potential functions of certain gut microbes, including the isolation of *Bacillus*, *Lysinibacillus* and *Clostridiales* species from cellulose and CMC media, indicating the ability to break down fibers. Cellulose degradation is common among several *Bacillus* species [72,73,74] and within the family *Ruminococcaceae* [75]. Several *Bacillus* species have previously been isolated from BSFL [17] and were also detected in this study in all replicates by amplicon sequencing, as well as in other studies [14,15,16,76]. However, the family *Bacillaceae* is one of the less prevalent taxa when BSFL are reared on chicken feed (relative abundance 0.3–2.1%, mean 1.2%), but might become more abundant when BSFL are fed on a high-fiber diet.

The diazotrophic growth of *Klebsiella* species on *Azotobacter* agar indicates the ability to fix atmospheric nitrogen, which is common within the genus *Klebsiella* [77,78]. By using media with low nutrient levels (such as 10% CASO agar or agar with CMC as the primary carbon source), we were able to isolate members of the phyla *Actinobacteria* and *Bacteroidetes* that have not been isolated from BSFL guts before. Based on amplicon sequencing data, these groups are generally among the less-abundant taxa, and are usually outcompeted by the more abundant families *Morganellaceae* and *Enterobacteriaceae* when rich growth media are used for bacterial isolation. Therefore, nutrient-poor media can help to selectively isolate *Actinobacteria* such as *Brevibacterium* species, which can tolerate low nutrient levels and survive starvation [79]. However, all the less-abundant genera of the phylum *Actinobacteria* were represented in at least four of the six BSFL gut samples, indicating their common occurrence in BSFL guts.

Strains belonging to the genera *Chryseobacterium*, *Glutamicibacter* and *Rummeliibacillus* were isolated from BSFL fed with keratin-rich or lignin-rich substrates [18]. *Chryseobacterium* and *Glutamicibacter* were not previously detected in BSFL by amplicon sequencing and were not isolated in this study or an earlier one [17], indicating their presence is diet-dependent.

Based on the pairwise comparison of 16S rRNA sequences, a large proportion (61.7%) of the isolated strains appeared to be redundant. However, genotyping by genomic fingerprinting revealed additional unique strains with potentially differing functions in the gut microbiome, thus reducing the overall redundancy to only 34.4%. Genotyping is also an important tool for the pre-selection of strains for further research, because excluding redundant strains from subsequent testing saves time and resources.

### 4.2. Antimicrobial Activity of the Gut Microbiome

The gastrointestinal tract is one of the most vulnerable access routes for insect pathogens [80]. Especially when BSFL are reared on waste products or decaying substrates, the host and its gut microbiome have to deal with strong bacterial pressure and have evolved corresponding defensive strategies. One example is the production of multiple AMPs with a broad activity spectrum by the insect immune system. Many such AMPs are induced by feeding on diets containing a high bacterial load, and contribute to the elimination of pathogens [36]. Another strategy is the establishment of a protective gut microbiome. However, the activity of BSFL gut bacteria against pathogens has not been investigated thus far. The inhibition zone assay is a rapid way to confirm the direct effect of gut microbes on pathogens. We found that 15 of the non-redundant bacterial isolates inhibited at least one of three tested pathogens. The inhibitory effect of the gut isolates observed in vitro might also occur in vivo when such pathogens enter the gut. This was supported by the absence of pathogens such as *Pseudomonas* sp., which were detected in the feed and the initial rearing phase but not in the guts of L5 larvae [6].

The genera *Alcaligenes*, *Morganella* and *Providencia* showed antimicrobial activity against pathogens in vitro and the last two are part of the BSFL core microbiome [6,13,16] indicating a protective role in the gut. *Bacillus velezensis* is known to produce several antimicrobial molecules [81], explaining the observed activity of the isolated *B. velezensis* strain against all three tested bacterial pathogens (including Gram-negative and Gram-positive species). Additional inhibitory mechanisms include iron scavenging by siderophores, which is common among the *Enterobacteriaceae* [82]. Several members of the family *Enterobacteriaceae* are known to protect insects against pathogens, including larvae of the burying beetle *Nicrophorus vespilloides* [83] and the desert locust *Schistocerca gregaria* [29]. *P. rettgeri* and *M. morganii* produce extracellular bacteriolytic enzymes that can degrade components of *E. coli* and *P. aeruginosa* cell walls [84,85]. The high relative abundance of *Morganella* (in both larval groups) and *Providencia* (in G1) may explain why common pathogens such as *Pseudomonas* and *E. coli* were scarce in BSFL gut samples in this study and our previous work [6]. However, for many of the BSFL gut bacterial isolates, the mechanism of pathogen-inhibition still has to be elucidated.

Extracts of whole BSFL inhibited *P. fluorescens* [36] and several other *Pseudomonas* species that act as plant pathogens [86], probably reflecting the effect of AMPs produced by the larvae. Ammonia production by BSFL increases the pH of the substrate and can also contribute to the elimination of certain pathogens, including *E. coli* and *Salmonella* species [7,87]. Moreover, the unique pH conditions in the BSFL gut may provide a selective barrier against certain pathogens [6,11,88].

The ability of BSFL to survive and thrive in environments with high pathogen loads is therefore likely to reflect the combined effect of multiple adaptations including AMPs and other effectors produced by the insect immune system, the protective microbiome that outcompetes pathogens and produces antimicrobial metabolites as well as the unique physiochemical conditions in the gut.

### 4.3. Possible Applications of BSFL Gut Bacterial Isolates

The antimicrobial compounds produced by insect-associated microbes, especially those protecting their hosts from severely contaminated environments, are potentially useful for drug development [32,89,90]. The bacterial isolates from BSFL guts in this study provide a large collection of strains from four phyla obtained under both aerobic and anaerobic cultivation conditions. Whereas previous studies focused only on the most abundant aerobic or facultative anaerobic BSFL-associated bacteria [18], we also isolated less-abundant taxa by using selective media, thus assembling a more diverse strain collection (26 genera and one novel genus in the order *Clostridiales*). In particular, members of the phylum *Actinobacteria* have rarely been isolated from BSFL guts, but we isolated representatives of 13 different species from six different families in this phylum.

To our knowledge, BSFL gut bacteria have yet to be exploited for medical, pharmaceutical or agricultural applications. Our initial tests have shown that several members of the gut microbiome can inhibit growth of pathogens in vitro. An in-depth screening of the BSFL culture collection, including a larger set of pathogens relevant in additional industrial sectors (e.g., food and feed, aquaculture, agriculture and medicine) may reveal further strains with useful antimicrobial properties. Some of these strains could be applied directly to BSFL feed substrates as probiotics, helping to reduce the pathogen load and maintain a healthy BSFL microbiome.

Using insects as feed in aquaculture is advantageous when compared to fishmeal not only for ecological reasons but also for sustainability [91,92] and animal health [93,94,95]. The extensive use of fishmeal brings dangers such as transmitting of fish-associated pathogens and other pollutants to farmed marine animals. BSFL show a similar composition of essential amino acids to fishmeal [2]. Therefore, BSFL meal can substitute large parts of fishmeal in diets for several finfish and ornamental fish species [96,97,98]. Administration of BSFL meal lead also to a significant colonization of the intestine by BSFL-associated bacteria in the Atlantic salmon. This has been shown by an increased relative abundance of the taxa *Actinomyces*, *Bacillus*, *Brevibacterium*, *Corynebacterium*, *Enterococcus*, *Oceanobacillus* and RsaHF231 [99], which have all been detected in this study in the BSFL guts in most of the replicates. Since RsaHF231 has not been documented in fish before, it likely originated from BSFL meal [99]. Furthermore, a beneficial effect of the BSFL diet on the fish gut microbiome has been suggested. Feeding BSFL meal to rainbow trout showed an increased relative abundance of the genus *Carnobacterium*, which is known for its probiotic effects (e.g., in vitro growth inhibition of pathogens and in vivo improvement of disease resistance) in salmonids [100]. *Carnobacteriaceae* have also been detected in the guts of BSFL in this study and by others [16,101]. These observations indicate that after ingestion, beneficial microbes form the BSFL gut can subsequently also colonize the fish intestine. Therefore, BSFL could possibly indirectly be used as probiotic for fish and other marine animals. By feeding beneficial microbes to the BSFL, its gut microbiome could be optimized especially for aquaculture applications and might help to control certain fish-pathogens. However, detailed studies are needed in order to test if BSFL-based feed may show probiotic effects in marine animals and will help to reduce the use of antibiotics in aquaculture.

Other strains in the culture collection may have the potential to degrade toxic components in feed [6], and could be added to certain industrial side-streams or waste products in order to remediate these substrates. Finally, BSFL gut bacterial isolates could be added to difficult-to-digest side-streams with a high content of lignocellulose, in order to enhance larval growth performance. In particular, endogenous (autochthonous) bacteria might not only pre-digest the substrate but will also colonize the gut and deploy their metabolic and probiotic activity within the gut, which probably would not be possible using laboratory strains [102,103,104]. *Bacillus* species (e.g., *B. subtilis* and *B. licheniformis*) isolated from BSFL conferred beneficial effects by promoting larval growth when added to feed [17,105]. In this study, we isolated various species of *Bacillaceae* (closely related to *B. velezensis*, *B. badius* and *Lysinibacillus macroides*) by using selective media containing cellulose and by pasteurization of the enrichment cultures. These strains can be tested for their hydrolytic activity and capacity to enhance larval growth, and may be useful for the industrial rearing of BSFL on side-streams with a high content of fiber.

## 5. Conclusions

We have carried out the first comprehensive study exploring the BSFL gut microbiome using both cultivation-independent and cultivation-dependent approaches. We found that the majority of the BSFL gut microbiome is composed of aerobic and facultative anaerobic bacteria, and we were able to cultivate the most abundant genera detected by amplicon sequencing. We were also able to cultivate several less-abundant genera and two obligate anaerobic strains. Genomic fingerprinting revealed redundant bacterial isolates, especially within the phyla *Proteobacteria* and *Bacteroidetes*. Initial tests with the remaining non-redundant isolates revealed several strains with the ability to inhibit *E. coli*, *S. aureus* and *P. aeruginosa*, and these may help to eliminate pathogens that enter the BSFL digestive system. Our study has also provided a valuable collection of unique bacterial isolates that can be screened for further beneficial properties, including the synthesis of bioactive natural products and useful enzymes (e.g., cellulases). The bacteria can be applied as probiotics and can be used for the pre-digestion of fiber-rich industrial side-streams to achieve more effective bioconversion by BSFL.

## Figures and Tables

**Table 1:** Activity of selected isolates against the pathogens *Staphylococcus aureus* (DSM 799), *Escherichia coli* K12 and *Pseudomonas aeruginosa* (DSM1117). Only isolates that showed activity are shown. Full clearance is indicated by +, partial clearance is indicated by (+) and no clearance is indicated by −. Values are means ± standard deviations of duplicate tests.

		*S. aureus*		*E. coli*		*P. aeruginosa*	
Strain No.	Closest Relative (Based on 16S rRNA Sequencing)	Inhibition	Zone of Inhibition (mm)	Inhibition	Zone of Inhibition (mm)	Inhibition	Zone of Inhibition (mm)
01-003	*Corynebacterium terpenotabidum*	−	−	−	−	(+)	1.50 ± 0.71
01-008	*Morganella morganii*	−	−	−	−	+	3.25 ± 0.35
01-018	*Alcaligenes faecalis*	+	2.75 ± 0.35	+	3.50 ± 0.00	−	−
01-027	*Providencia rettgeri*	−	−	−	−	(+)	3.00 ± 0.00
01-035	*Providencia rettgeri*	−	−	−	−	(+)	2.50 ± 0.00
01-040	*Wohlfahrtiimonas larvae*	(+)	1.00 ± 0.00	−	−	+	2.00 ± 0.00
01-052	*Providencia rettgeri*	−	−	−	−	(+)	3.00 ± 0.00
01-053	*Providencia rettgeri*	−	−	−	−	(+)	2.00 ± 0.00
01-054	*Klebsiella aerogenes*	−	−	+	3.75 ± 0.35	−	−
01-060	*Providencia rettgeri*	−	−	−	−	(+)	3.00 ± 0.00
01-079	*Providencia rettgeri*	−	−	−	−	(+)	2.00 ± 0.00
01-105	*Klebsiella aerogenes*	−	−	+	3.25 ± 0.35	−	−
01-115	*Alcaligenes faecalis*	+	2.00 ± 0.00	−	−	−	−
01-124	*Alcaligenes faecalis*	−	−	+	2.75 ± 0.35	+	3.50 ± 0.71
01-149	*Bacillus velezensis*	+	2.75 ± 0.35	+	1.50 ± 0.00	+	5.25 ± 1.06

## Data Availability

The datasets presented in this study can be found in online repositories. The 16S rRNA gene sequences of the isolates have been submitted to GenBank (http://www.ncbi.nlm.nih.gov) and can be accessed under the numbers MZ413999-MZ414160. The amplicon data can be accessed at NCBI under the BioProject PRJNA739514.

## Supplementary Materials

The following are available online at https://www.mdpi.com/article/10.3390/microorganisms9081642/s1, Table S1: Interactive Excel spreadsheet providing details of the bacterial community composition based on 16S rRNA amplicon sequencing classified using SILVA 138.1 and the NCBI nucleotide databases, Table S2: Classification the bacterial isolates based on Sanger sequences using SILVA 138.1 and the NCBI nucleotide databases, Table S3: Pairwise comparison of 16S rRNA sequences similarities of isolates and closest related type strains belonging to the phyla (A) *Proteobacteria* (B) *Firmicutes* (C) *Bacteroidetes* and (D) *Actinobacteria*, Figure S1: Dendrogram constructed from BOX-PCR banding patterns showing genetic relatedness of 157 isolates, Figure S2: Dendrogram constructed from GTG5-PCR banding patterns showing genetic relatedness of three isolates.
