# Supplementary figures and images for: Culture-Independent and Culture-Dependent Characterization of the Black Soldier Fly Gut Microbiome Reveals a Large Proportion of Culturable Bacteria with Potential for Industrial Applications
Source: Microorganisms. 2021 Jul 31;9(8):1642. doi: 10.3390/microorganisms9081642 (PMC8398798; doi:10.3390/microorganisms9081642)

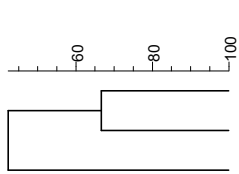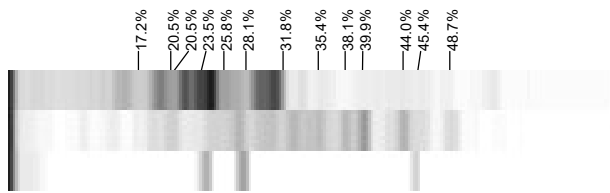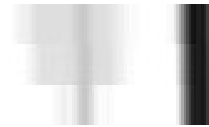

01-049  
01-098  
01-048

Supplement: Supplementary file 1 [file microorganisms-09-01642-s001.zip › Figure S2.pdf]

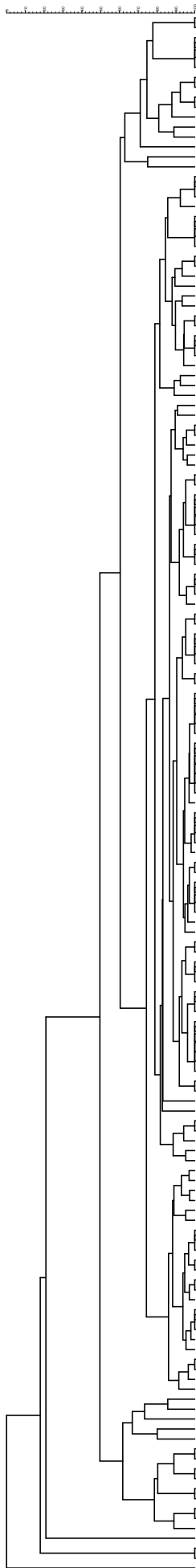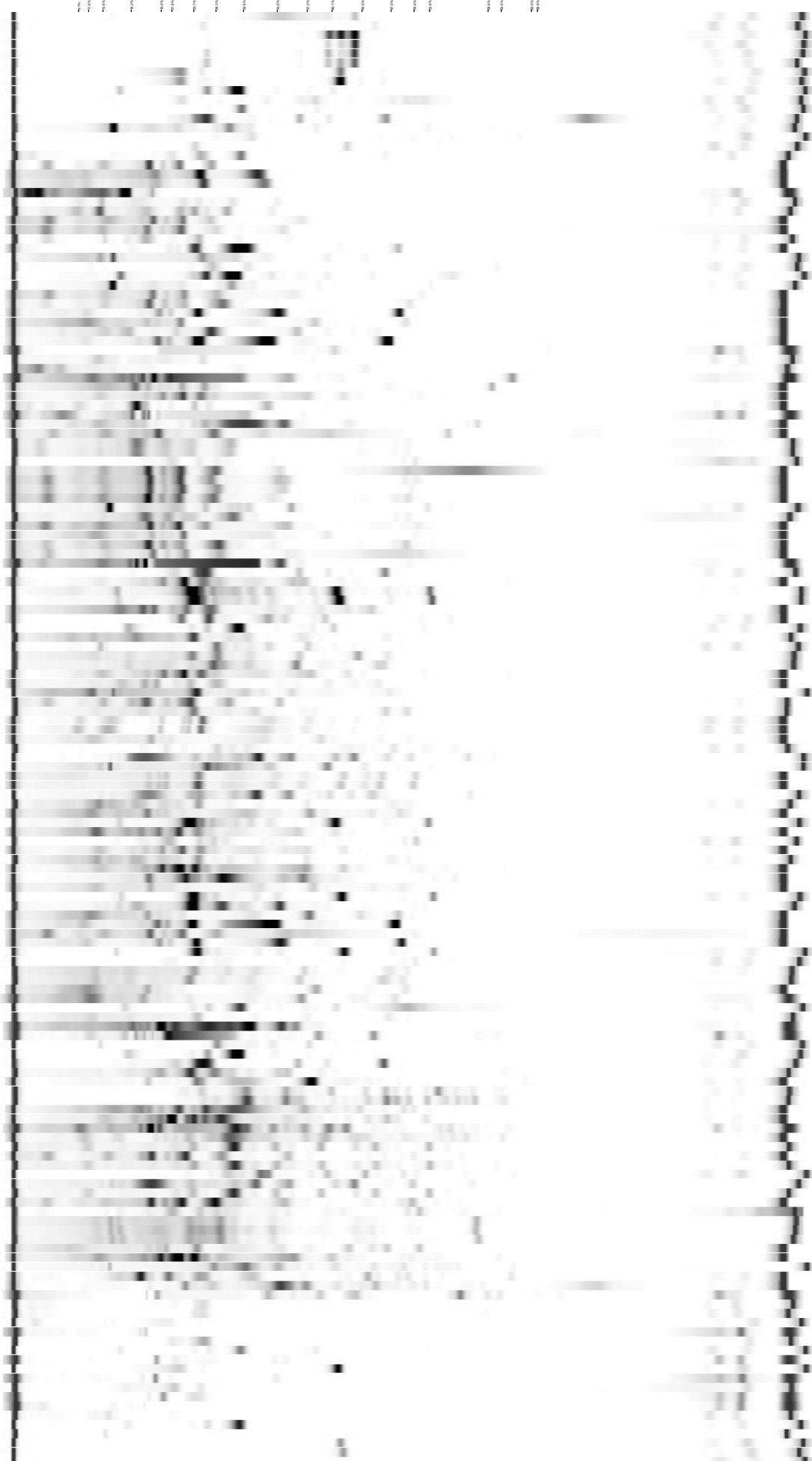

01-025  
01-045  
01-007  
01-017  
01-022  
01-050  
01-014  
01-026  
01-010  
01-030  
01-020  
01-051  
01-061  
01-005  
01-048  
01-093  
01-151  
01-168  
01-169  
01-102  
01-059  
01-052  
01-112  
01-167  
01-073  
01-150  
01-039  
01-047  
01-016  
01-098  
01-137  
01-176  
01-114  
01-139  
01-154  
01-129  
01-040  
01-072  
01-097  
01-094  
01-105  
01-119  
01-149  
01-085  
01-109  
01-131  
01-070  
01-071  
01-052  
01-170  
01-142  
01-164  
01-165  
01-044  
01-084  
01-123  
01-110  
01-175  
01-144  
01-008  
01-096  
01-130  
01-028  
01-029  
01-156  
01-171  
01-033  
01-078  
01-043  
01-063  
01-064  
01-115  
01-157  
01-001  
01-099  
01-101  
01-107  
01-108  
01-140  
01-096  
01-018  
01-019  
01-121  
01-122  
01-038  
01-083  
01-148  
01-036  
01-128  
01-053  
01-120  
01-081  
01-154  
01-124  
01-163  
01-034  
01-052  
01-161  
01-103  
01-155  
01-106  
01-012  
01-035  
01-074  
01-076  
01-138  
01-162  
01-023  
01-089  
01-177  
01-090  
01-024  
01-032  
01-056  
01-091  
01-117  
01-086  
01-092  
01-118  
01-126  
01-113  
01-127  
01-087  
01-166  
01-088  
01-003  
01-042  
01-089  
01-152  
01-060  
01-054  
01-057  
01-067  
01-111  
01-116  
01-002  
01-174  
01-125  
01-066  
01-068  
01-095  
01-027  
01-015  
01-077  
01-006  
01-173  
01-004  
01-172  
01-075  
01-178  
01-079  
01-080  
01-031  
01-100  
01-021  
01-037  
01-013

Supplement: Supplementary file 1 [file microorganisms-09-01642-s001.zip › figure_S1.pdf]
